# Supplementary material for: The Viromes of Mosquitoes from the Natural Landscapes of Western Siberia
Source: Viruses. 2023 Sep 8;15(9):1896. doi: 10.3390/v15091896 (PMC10537626; doi:10.3390/v15091896)
Supplement: Supplementary file 1 [file viruses-15-01896-s001.zip › viruses-2530126-supplementary.pdf]

viruses

Supplementary Materials

The Viromes of Mosquitoes from the Natural Landscapes of Western Siberia

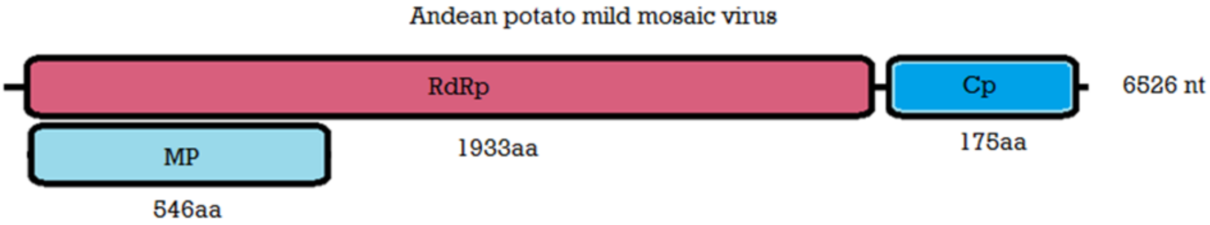

(**a**)

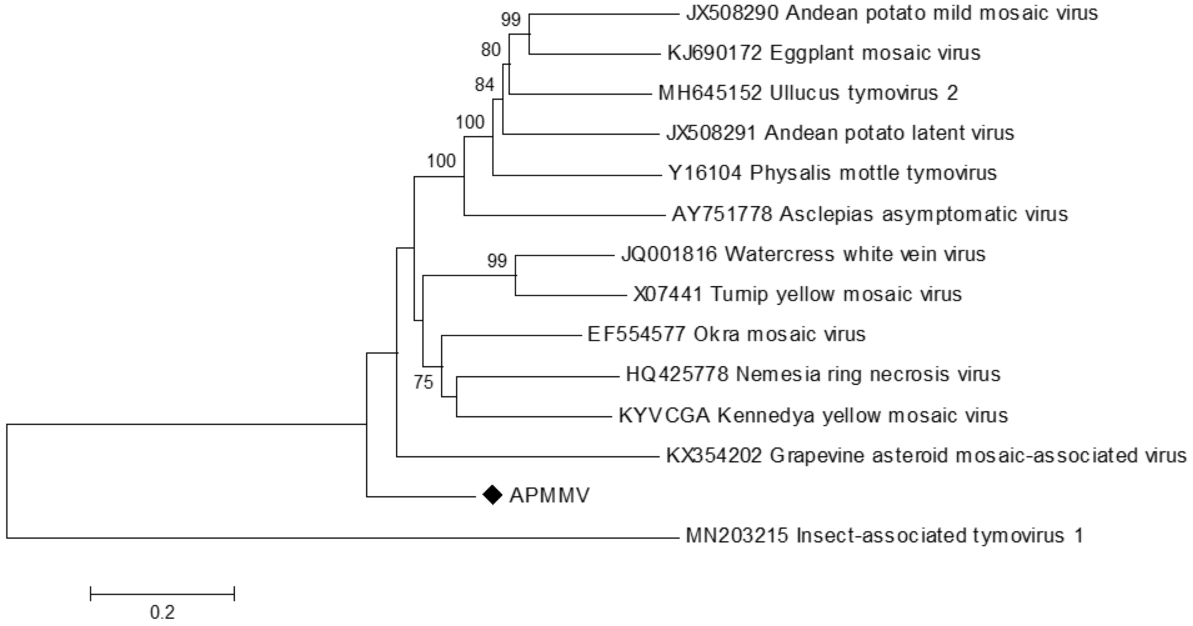

(**b**)

Figure S1. Andean potato mild mosaic virus (APMMV) as a novel Inya insect-associated virus 1 and 2. The APMMV are close to insect-associated tymovirus 1 and 2 (near 60% identity). (*Viruses; Riboviria; Orthornavirae; Kitrinoviricota; Alsuviricetes; Tymovirales*; unclassified *Tymovirales*.). (**A**) The scheme of genome organization for Insect-associated tymovirus 1; (**B**) The phylogenetic trees for RNA viruses for selected *Tymoviralis* and APMMV.

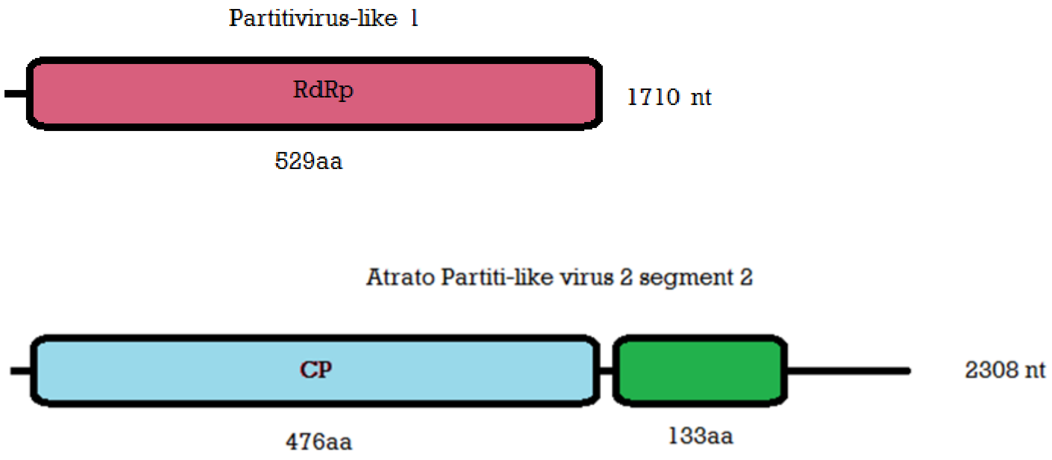
Partitivirus-like 1
RdRp
1710 nt
529aa
Atrato Partiti-like virus 2 segment 2
CP
2308 nt
476aa
133aa

(a)

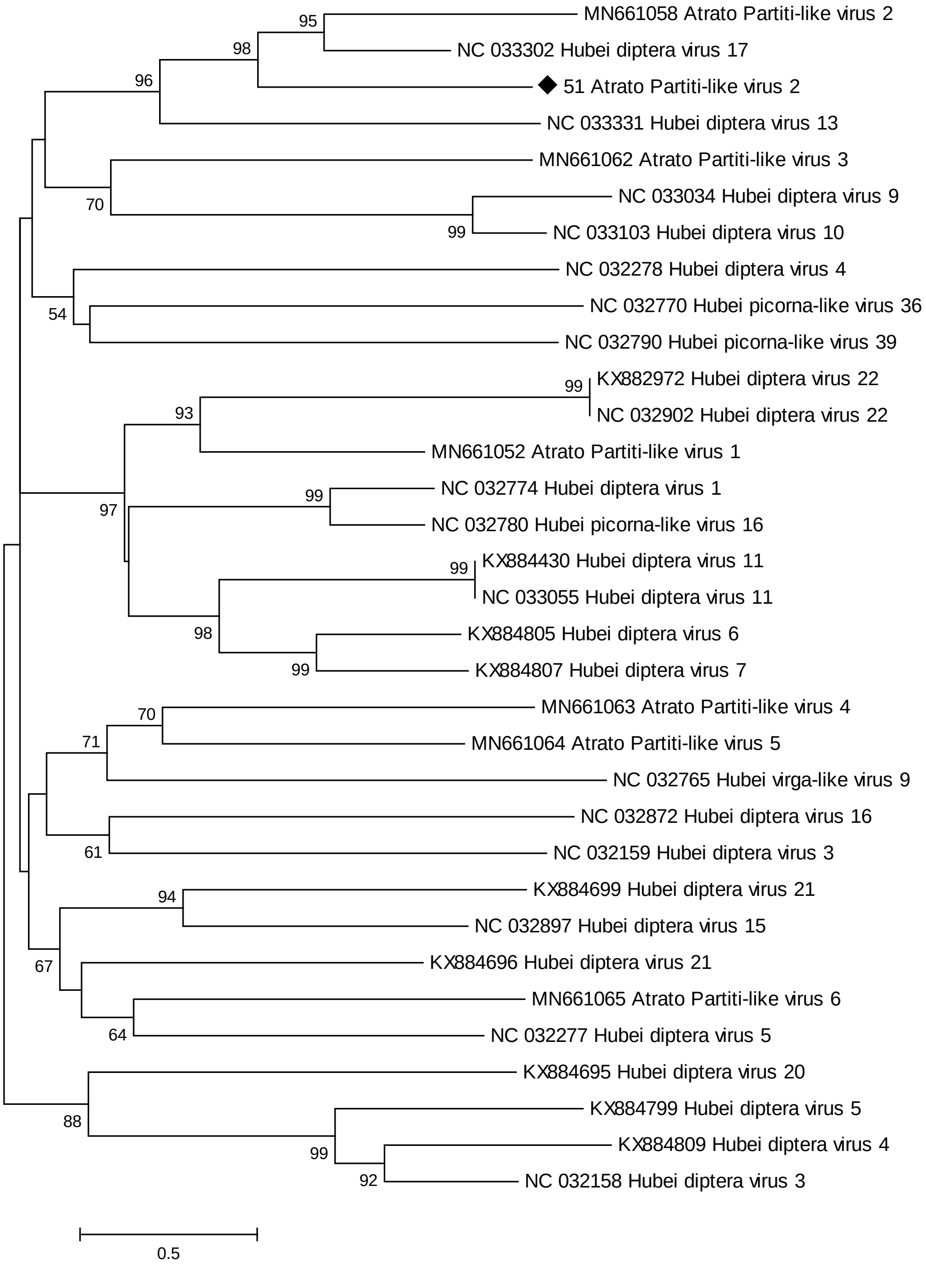
MN661058 Atrato Partiti-like virus 2
NC 033302 Hubei diptera virus 17
51 Atrato Partiti-like virus 2
NC 033331 Hubei diptera virus 13
MN661062 Atrato Partiti-like virus 3
NC 033034 Hubei diptera virus 9
NC 033103 Hubei diptera virus 10
NC 032278 Hubei diptera virus 4
NC 032770 Hubei picorna-like virus 36
NC 032790 Hubei picorna-like virus 39
KX882972 Hubei diptera virus 22
NC 032902 Hubei diptera virus 22
MN661052 Atrato Partiti-like virus 1
NC 032774 Hubei diptera virus 1
NC 032780 Hubei picorna-like virus 16
KX884430 Hubei diptera virus 11
NC 033055 Hubei diptera virus 11
KX884805 Hubei diptera virus 6
KX884807 Hubei diptera virus 7
MN661063 Atrato Partiti-like virus 4
MN661064 Atrato Partiti-like virus 5
NC 032765 Hubei virga-like virus 9
NC 032872 Hubei diptera virus 16
NC 032159 Hubei diptera virus 3
KX884699 Hubei diptera virus 21
NC 032897 Hubei diptera virus 15
KX884696 Hubei diptera virus 21
MN661065 Atrato Partiti-like virus 6
NC 032277 Hubei diptera virus 5
KX884695 Hubei diptera virus 20
KX884799 Hubei diptera virus 5
KX884809 Hubei diptera virus 4
NC 032158 Hubei diptera virus 3
95
98
96
70
99
54
99
93
97
99
99
98
99
70
71
61
94
67
64
88
99
92
0.5

(**b**)

Figure S2. A novel virus with a 51% identity to Atrato Partiti-like virus 2 was detected in the Anopheles messeae pool and referred to as the Krahall insect-associated virus. (**A**) The scheme of genome organization for *Partitivirus*. (**B**) The phylogenetic relationship of *Partitivirus* with Atrato Partiti-like virus 2, based on the alignment of the RdRp and the putative scheme for the partitivirus genome.

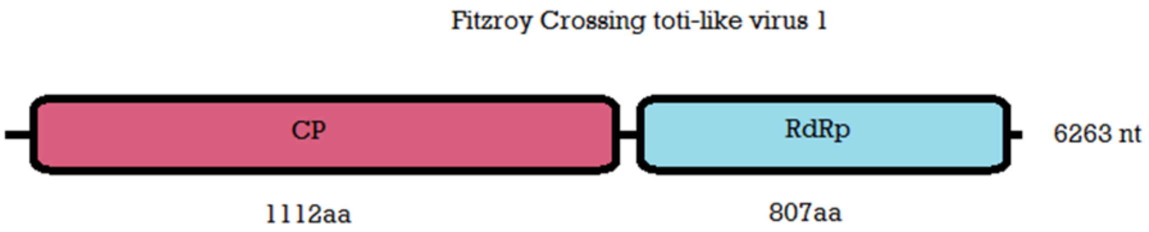

Figure S3. The scheme of genome organization for the Fitzroy Crossing toti-like virus 1.

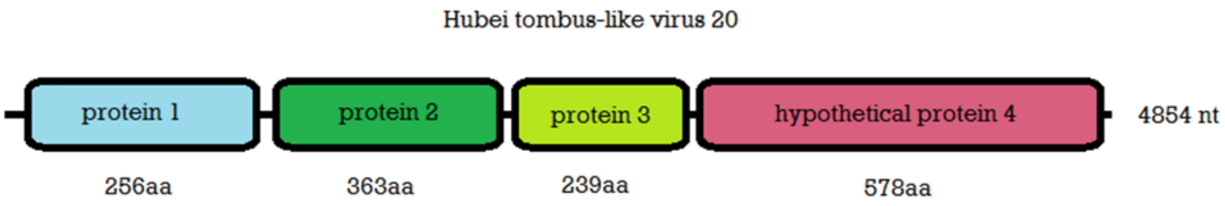

Figure S4. The scheme of genome organization for the Tombus-like virus. .

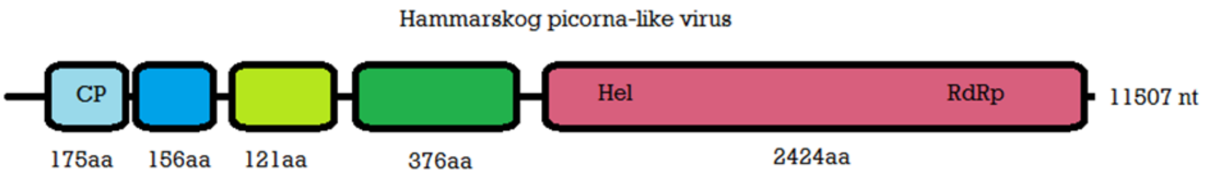

Figure S5. The genome organization for the picorna-like virus.

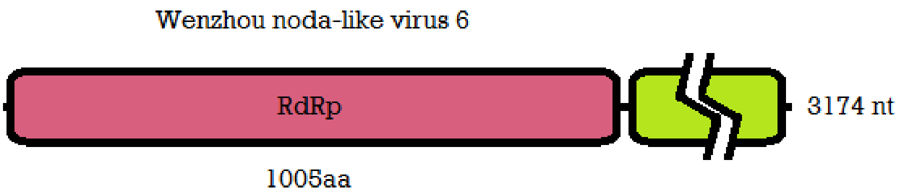

Figure S6. The genome organization for the nodaviruses (ssRNA).

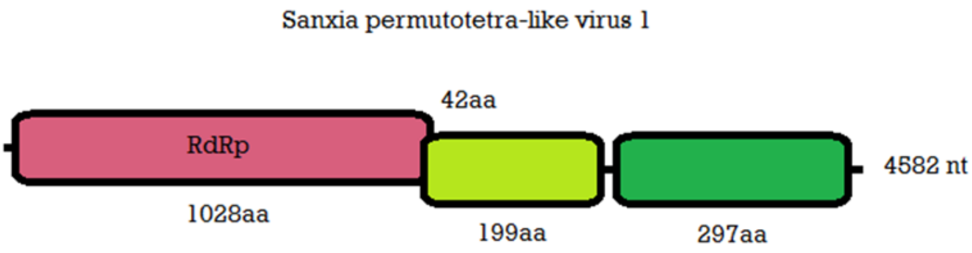

Figure S7. The genome organization for the permutotetraviruses.
